# Impact of Sociodemographic Characteristics, Lifestyle, and Obesity on Coexistence of Diabetes and Hypertension: A Structural Equation Model Analysis amongst Chinese Adults

**DOI:** 10.1155/2021/4514871

**Published:** 2021-10-25

**Authors:** Wenwen Wu, Jie Diao, Jinru Yang, Donghan Sun, Ying Wang, Ziling Ni, Fen Yang, Xiaodong Tan, Ling Li, Li Li

**Affiliations:** ^1^Institute for Evidence-Based Nursing, Renmin Hospital, Hubei University of Medicine, Shiyan 442000, China; ^2^School of Public Health, Hubei University of Medicine, Shiyan 442000, China; ^3^Center for Environment and Health in Water Source Area of South-to-North Water Diversion, Hubei University of Medicine, Shiyan 442000, China; ^4^School of Engineering, University of Glasgow, Glasgow G12 8QQ, UK; ^5^Tongji Medical College, Huazhong University of Science and Technology, Wuhan 430030, China; ^6^Department of Nosocomial Infection Management, Wuhan University Zhongnan Hospital, Wuhan 430071, Hubei, China; ^7^School of Medicine, Hangzhou Normal University, Hangzhou 311121, China; ^8^College of Nursing, Hubei University of Chinese Medicine, Wuhan 430065, China; ^9^School of Health Sciences, Wuhan University, Wuhan 430071, China; ^10^Nursing Department, Dongfeng Hospital, Hubei University of Medicine, Shiyan 442000, China

## Abstract

**Background:**

In general, given the insufficient sample size, considerable literature has been found on single studies of diabetes and hypertension and few studies have been found on the coexistence of diabetes and hypertension (CDH) and its influencing factors with a large range of samples. This study aimed to establish a structural equation model for exploring the direct and indirect relationships amongst sociodemographic characteristics, lifestyle, obesity, and CDH amongst Chinese adults.

**Methods:**

A cross-sectional study was conducted in a representative sample of 25356 adults between June 1, 2015, and September 30, 2018, in Hubei province, China. Confirmatory factor analysis was initially conducted to test the latent variables. A structural equation model was then performed to analyse the association between latent variables and CDH.

**Results:**

The total prevalence of CDH was 2.8%. The model paths indicated that sociodemographic characteristics, lifestyle, and obesity were directly associated with CDH, and the effects were 0.187, 0.739, and 0.353, respectively. Sociodemographic characteristics and lifestyle were also indirectly associated with CDH, and the effects were 0.128 and 0.045, respectively. Lifestyle had the strongest effect on CDH (*β* = 0.784, *P* < 0.001), followed by obesity (*β* = 0.353, *P* < 0.001) and sociodemographic characteristics (*β* = 0.315, *P* < 0.001). All paths of the model were significant (*P* < 0.001).

**Conclusion:**

CDH was significantly associated with sociodemographic characteristics, lifestyle, and obesity amongst Chinese adults. The dominant predictor of CDH was lifestyle. Targeting these results might develop lifestyle and weight loss intervention to prevent CDH according to the characteristics of the population.

## 1. Introduction

Hypertension (HT) and diabetes mellitus (DM) are major noncommunicable diseases (NCDs), which are important global public health problems [1]. Although they are two distinct medical conditions, they frequently coexist, leading to additive increases in the risk of cardiovascular diseases [2]. Research has reported that amongst patients with DM, HT prevalence is 82.1% in American adults [3], 54.6% in Korean adults [4], and 49.9% to 60.6% in Chinese outpatients. A study conducted at a hospital in Delhi, India, has shown that the prevalence of coexistence of DM and HT (CDH) is 31% [5].

Many of the factors associated with HT and DM are the same, such as sociodemographic characteristics. The results of some studies have found that both diseases are common amongst males, aged, unmarried, and those who are less educated, and those with lower income are also at higher risk [6–10]. However, an earlier study conducted in China has found no association between education and prevalence of HT [11]. The relationship between social demographic characteristics and CDH needs to be further explored.

Lifestyle has also been shown to have a significant effect on the risk of HT and DM. Several studies have shown that drinking alcohol, smoking, physical inactivity, and insufficient intake of calcium, magnesium and fish fatty acids can increase the prevalence of both diseases [12–16]. However, not all studies have come to the same conclusion. For example, a cohort study in Denmark has shown that alcohol consumption can reduce the risk of DM [17]. At present, few researchers have explored the relationship between lifestyle and CDH.

In addition, numerous studies have shown that obesity is a well-established risk factor for DM and HT [18, 19]. Many anthropometric indexes of obesity have been identified, such as body mass index (BMI), waist circumference (WC), and waist-to-height ratio (WHR). BMI is a more widely used measure to diagnose obesity than WC and WHR [20]. One study conducted by Okosun et al. has found that obesity is associated with increased risk of CDH in white, black, and Hispanic men and women [21]. Moreover, they have discovered the independent roles of these indices. However, previous studies have shown racial differences in the associations between these anthropometric measures and the prevalence of HT or DM [22–24]. Given these ethnic differences, the influence of obesity on the prevalence of CDH amongst Chinese adults and the mechanism of different anthropometric measures remain unclear.

In general, given the insufficient sample size, considerable literature has been found on single studies of DM and HT, and few studies have been found on CDH and its influencing factors with large range of samples. In addition, is there any relationship amongst sociodemographic characteristics, lifestyle, obesity, and the prevalence of CDH? If so, how do they affect CDH? These are questions that need to be studied urgently.

The population aging process is ahead of the economic development process, which makes the risks and challenges faced by China more severe. The health of adult is related not only to individuals and families, but also to the social stability and economic growth. Relevant information on the prevalence and determinants of CDH amongst adult samples is crucial for establishing goals for effective and targeted intervention programs.

Therefore, we conducted a large representative sampling study on Chinese adults to fill the abovementioned knowledge gaps, describe the prevalence of CDH amongst adults, and explore the effect of demographic sociological characteristics, lifestyle, and obesity on CDH and the specific impact path by using a structural equation model (SEM).

## 2. Materials and Methods

### 2.1. Study Design and Participants

A population-based cross-sectional questionnaire survey was conducted in 11 districts of Hubei Province, China, from June 1, 2015, to September 30, 2018. The participants in each district were selected using a multistage stratified random sampling strategy with the assistance of Centers for Disease Control and Prevention in each administrative district. Firstly, six towns were randomly selected from each district. Secondly, six communities or villages were randomly selected from each town. In the third stage, at least 60 families were randomly selected from each community or village. Finally, one resident over 18 years old was randomly selected from each family. Residents were required to meet the following inclusion criteria: (1) age ≥18 years old and (2) informed and agreed to participate in the survey. People with allopathy or consciousness disorder or severe cognitive impairment were excluded from this study.

The formula *n* = *Z*^2^*PQ*/*d*^2^ was used to calculate the sample size (where *Z* = 1.96 at 95% confidence interval; *P* = prevalence of CDH; *Q* = 1 − *P*; and *d* = absolute allowable error) [25]. For this study, the prevalence of CDH was set at 5.2% based on a previous study [26], and *d* = 0.1. Thus, the sample size was at least 2083 in each district. Of the 29396 questionnaires distributed, 25356 were usable for an overall response rate of 86.3% (4,040 individuals with missing data were excluded). Participation was voluntary, and no incentives were offered in return for participation. The study was performed in accordance with the Declaration of Helsinki. Ethical approval was obtained from the ethics board of Hubei University of Medicine (no. 2020-TH-058).

### 2.2. Measures

A self-administered multi-section questionnaire, which was designed on the basis of the contents of the Monitoring of Chronic Diseases and Their Risk Factors (2013) working Manual issued by the Chinese Center for Disease Control and Prevention [27], was used to collect the following data through interviewing the participants at the participant's homes.

Sociodemographic indicators included age (years), gender, educational status (illiterate or some primary school, primary school graduate or some junior high school, junior high school graduate or some senior high school, senior high school graduate or some college, and college graduate or above), marital status (single, married, and divorced/widowed/separated), occupation, and per capita family monthly income (PCFMI; <1000 RMB, 1000–1500 RMB, 1500–2000 RMB, and ≥2000 RMB).

With regard to lifestyle, participants were invited to answer questions on their current status of smoking (active or passive smoking), alcohol drinking, psychosocial work intensity, physical exercise, and daily static behaviour time (including sitting work, learning, reading, watching television, using computer, rest and other static behaviour time but not sleeping time, salt consumption, and oil consumption). Smoking status was determined by asking participants, “Are you currently a smoker? Are you a passive smoker?” Participants who replied that they smoked “everyday” or on “some days” or “they were passive smoker” were classified as current smokers. Those who replied “not a smoker or a passive smoker” were classified as noncurrent smokers.

Health knowledge related to chronic diseases was measured by asking the following four questions: (1) “whether eating more salt will affect health? (persons who answered “no” or “do not know” were classified as they do not know the knowledge);” (2) “do you know the standard of daily salt intake per person? (persons who answered “no” or “do not know” were classified as they do not know the knowledge);” (3) “do you know the standard of daily oil intake per person? (persons who answered “no” or “do not know” were classified as they do not know the knowledge);” (4) “do you know the criteria for people at high risk for chronic disease?” (the criteria are as follows: blood pressure of 130–139 mmHg/85–89 mmHg; current smokers; fasting blood glucose was 6.1 ≤ EBG < 7.0 mmol/L; serum total cholesterol was 5.2 ≤ TB < 6.2 mmol/L; participants who correctly answered at least one of these criteria were classified as “yes”).

We also measured WC and the actual height and weight of all respondents using an ultrasonic height sensor, from which BMI and WHtR were computed. BMI was divided into four categories: thin (<18.5 kg/m^2^), moderate (18.5 kg/m^2^ to 23.9 kg/m^2^), overweight (24.0 kg/m^2^ to 26.9 kg/m^2^), and obese (>27.0 kg/m^2^) [28, 29]. WC was divided into two categories: male ≥ 90 cm, females ≥ 85 cm [30].

The blood pressure of the subjects in the sitting position was measured three times with a mercury sphygmomanometer, and the average was used as the final value. HT was defined as SBP at least 140 mmHg, DBP at least 90 mmHg, current treatment with antihypertensive medication, or a self-reported diagnosis of HT [31]. Participants with fasting plasma glucose > 7.0 mmol/L or 2 h plasma glucose > 11.1 mmol/L after oral glucose tolerance test or those who were receiving antidiabetic medications were diagnosed with DM [32].

### 2.3. Pilot Study

A pilot quantitative study with 40 participants was conducted to improve the comprehensibility of the questionnaire. The pilot participant responses were then analysed for clarity, understandability, and applicability of the questionnaire. Internal consistency of the questionnaire was calculated by Cronbach's alpha and was found to be equal to 0.78, which was considered acceptable.

### 2.4. Data Collection Procedure

A questionnaire survey was undertaken through face-to-face interviews. Those who meet the inclusion criteria were informed of the study and asked if they were willing to participate, establishing informed consent. The questionnaire was completed independently and anonymously by targeted individuals to protect the privacy of participants. Participants were interviewed by trained interviewers if they requested assistance completing the survey (e.g., participants who have dyslexia). Finally, completed questionnaires were checked by qualified investigators (i.e., graduate students specifically trained to perform data collection for this study) to ensure the completeness of the questionnaires with immediate follow-up of participants who needed further information.

### 2.5. Quality Control

After the pretest and reasonable adjustment to the questionnaire measures, a formal questionnaire for study was formed. We asked our targeted individuals to answer the questionnaires anonymously, with no mention of personal details, and to fill out the survey questions independently based on their actual situation. Training was provided to all of the investigators and supervisors prior to the survey. During the implementation of the survey, we also made a clear explanation of the purpose and significance of this study to participants. The finished questionnaires were checked by supervisors to ensure the effectiveness of the questionnaires. The supervisors randomly checked 5% of the respondents in the survey sites, and the consistency rate of questionnaire filling should be greater than 95%.

### 2.6. Statistical Analysis

**Figure 1:**
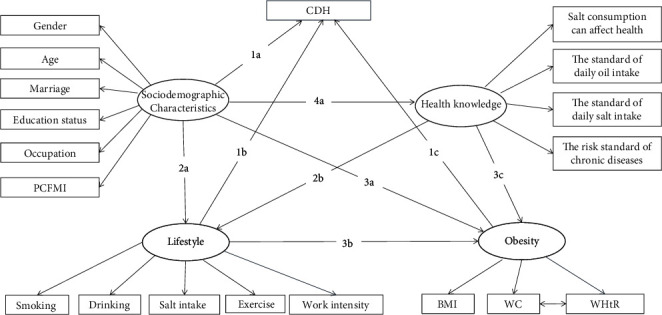
Base structural equation model.

Data were entered using Microsoft Excel. SPSS V.17.0 (SPSS, Chicago, IL, USA) was used to conduct a descriptive analysis of participants' general characteristics. Description was obtained using frequency and proportion. The reference 25th (*P*_25_), 50th (*P*_50_), and 75th (*P*_75_) percentiles were constructed for WC. Differences in CDH prevalence by general characteristic variables were assessed using *χ*^2^ test. The correlation matrix of the study variables was examined using Pearson's correlation coefficients. In this study, the multicollinearity assessment was conducted using the variance inflation factor (VIF), which is superior to check bivariate correlations [33]. A common rule is that VIF values ≥10 indicate the presence of multicollinearity [34]. Confirmatory factor analysis (CFA) was first conducted to test whether the latent variables could be well reflected by the involved observational variables. Based on the results of CFA and an exhaustive literature review, we proposed the research model (Figure 1) of this study and seven hypotheses. These hypotheses are presented as follows:

  Hypothesis 1a: CDH is influenced by sociodemographic characteristics
  Hypothesis 1b: CDH is influenced by lifestyle
  Hypothesis 1c: CDH is influenced by obesity
  Hypothesis 2a: lifestyle is influenced by sociodemographic characteristics
  Hypothesis 2b: lifestyle is influenced by health knowledge
  Hypothesis 3a: obesity is influenced by sociodemographic characteristics
  Hypothesis 3b: obesity is influenced by lifestyle
  Hypothesis 3c: obesity is influenced by health knowledge
  Hypothesis 4a: health knowledge is influenced by sociodemographic characteristics

Mplus version 7.4 was used to conduct a SEM for analysing the complexity of associations amongst obesity, lifestyle, health knowledge, sociodemographic characteristics, and CDH. SEM is a statistical method that takes a confirmatory approach to the analysis of a structural theory [35]. SEM has been widely used in the research field of social psychology and behavioural medicine. It has also been increasingly used in the study of effective interventions for NCDs [36–38]. SEM is superior to the traditional regression method. Weighted least squares with the mean and variance-adjusted methods were used for the parameter estimation of CFA and SEM. All *P* values were two-sided, with values <0.05 considered as statistically significant. The goodness of fit of the SEM was examined using the following four goodness-of-fit indices: maximum likelihood chi-square (*χ*^2^) and degrees of freedom (d*f*), Tucker–Lewis index [39], comparative fit index (CFI) [40], and the root mean square error of approximation (RMSEA) [41]. The value of *χ*^2^ was susceptible to the sample size, which was statistically significant for model with large sample size [42]. An acceptable model fit was defined by the following criteria: TLI (≥0.90), CFI (≥0.90), and RMSEA (≤0.08) [43].

This is the base model describing the complex relationships between all variables. “*X* ⟶ *Y*” means *X* influenced *Y*, “*X* ↔ *Y*” means *X* and *Y* influenced each other; CDH: coexistence of diabetes and hypertension; PCFMI: per capita family monthly income; BMI: body mass index; and WC: waist circumference.

## 3. Results

### 3.1. Participants' Demographic Characteristics

In this study, the prevalence of CDH amongst the adults was 2.8%. The general sociodemographic characteristics of participants are shown in Table 1. Amongst the 25,356 subjects, more than half were females (51.8%). The largest age group were composed of individuals aged 18–39 years (40%), followed by those aged 40–59 years (34.4%). 84.1% of the participants (21,328) were married. Approximately three-fifths of the respondents (59.26%) had a junior high school education or higher. Many respondents worked in businesses or services (35.3%) and had PCFMI ranging from 1,500 yuan to 2,000 yuan (RMB, 34.1%). The majority of the respondents were smoking or passively smoking (82.7%), drinking (73.7%), having a low work intensity (49.3%), having less than 4 h of static activity per day (64.8%), having awareness of the health effects of salt intake (63%), having salt intake of >18 g per day (53.4%), not engaging in physical exercise (80.9%), and having no knowledge of the standard of daily salt intake per person (79.2%). Only 20.8% and 18.0% of the participants knew the standards of daily salt and oil intake. Less than a third of the participants were aware of the risk standard of chronic diseases (27.8%). The largest BMI group was 18.5–23.9 kg/m^2^ (59.2%), and the majority of participants had a normal WC (53.4%). The participants were classified into four groups based on WHtR: <*P*_25_, *P*_25_ to <*P*_50_, *P*_50_ to <*P*_75_, and ≥*P*_75_, accounting for 25.3%, 24.9%, 25.8%, and 24%, respectively. The distribution of participant's general characteristics differed with regard to the prevalence of CDH (*P* < 0.05). The highest VIF value was 3.04, indicating that there was no evidence of multicollinearity in this study.

### 3.2. SEM Analysis of Obesity, Lifestyle, Health Knowledge, Sociodemographic Characteristics, and CDH

**Figure 2:**
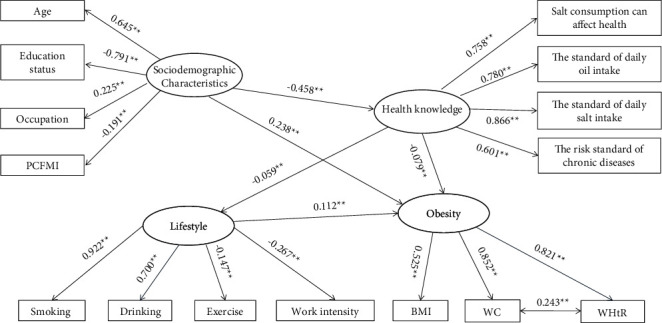
The measurement model of latent variables. Four latent variables and 15 manifest variables are connected by significant paths. *Note*. ^*∗∗*^*P* < 0.001 (two-tailed test). All path coefficients shown were standardized. PCFMI: per capita family monthly income; BMI: body mass index; WC: waist circumference; and WHR: waist-to-height ratio.

All hypothetical latent variables, namely, sociodemographic characteristics, lifestyle, obesity and health knowledge, were specified in the measurement model. Observational variables with insignificant paths or low factor loadings were deleted from the initial measurement model to determine the most parsimonious model. After preliminary data analysis, 15 observational variables were retained, and their assignment is illustrated in Supplementary Table 1. The correlation matrix of the study variables is presented in Table 2. Many of the variables showed significant correlations.  Figure 2 shows the final measurement model. Sociodemographic characteristics, lifestyle, obesity, and health knowledge were assigned to four, four, three, and four observational variables, and the factor loadings ranged from 0.191 to 0.791, 0.147 to 0.922, 0.525 to 0.852, and 0.601 to 0.866, respectively. All these factor loadings were significant (*P* < 0.05). Amongst the variables, sociodemographic characteristics displayed the strongest effect on health knowledge (*β* = −0.458); health knowledge had the weakest effect on lifestyle (*β* = −0.059). All the correlation paths were significant (*P* < 0.01). The measurement model well fitted to the data. The goodness-of-fit indices were as follows: *χ*^2^ = 9051.550, d*f* = 84, *P* < 0.001, CFI = 0.981, TLI = 0.977, and RMSEA = 0.065 (95% confidence intervals [CI]) = 0.064–0.066).

**Figure 3:**
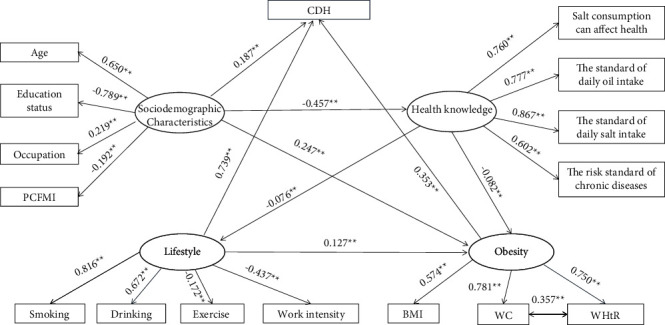
The structural equation model of sociodemographic characteristics, lifestyle, obesity, health knowledge, and HDC. *Note*. ^*∗∗*^*P* < 0.001 (two-tailed test). All path coefficients shown were standardized. CDH: coexistence of diabetes and hypertension; PCFMI: per capita family monthly income; BMI: body mass index; WC: waist circumference; and WHR: waist-to-height ratio.

SEM was used to explore the internal relationships amongst the latent variables, namely, obesity, lifestyle, health knowledge, and sociodemographic characteristics, and their impact on CDH. The base model showed poor model fit, with RMSEA failing to reach the cut-off criteria. The model fit improved with the reduction of mediators. As shown in Figure 3, the final structural model showed acceptable fit with the data: *χ*^2^ = 10573.237, d*f* = 96, *P* < 0.001, CFI = 0.978, TLI = 0.973, and RMSEA = 0.066 (95% CI = 0.065–0.067). As shown in the model, all indicator variables, which were hypothesised as predictors, were significantly related to their respective latent factors, *P* < 0.05. Lifestyle had the strongest direct effect on CDH (*β* = 0.739, *P*=0.017), followed by obesity (*β* = 0.353, *P*=0.020) and sociodemographic characteristic (*β* = 0.187, *P*=0.018). Obesity mediated effects on the association amongst health knowledge, sociodemographic characteristic, lifestyle, and CDH. The indirect effect of sociodemographic characteristic on CDH was the greatest, followed by health knowledge and lifestyle (Table 3). Except for “hypothesis 2a,” all of the hypotheses were supported. Overall, the model accounted for approximately 82.8% of the variance in CDH (*R*^2^ = .828, *P* < 0.001).

## 4. Discussion

This study is the first to explore the structural relationships of CDH prevalence with the possible associated factors amongst the Chinese adults. One of the strengths of this study is that we used SEM in data analysis, which is a powerful tool for developing a complex and sophisticated theoretical model [44]. SEM can enhance our understanding of the relationships amongst multiple factors, such as the relative contributions of anthropometric indexes of obesity and other factors related to CDH and the correlations between obesity and other factors. Furthermore, data of this study were drawn from a large-scale sampling survey, a large representative data set of adults currently available in China.

Our study found that the prevalence of CDH was lower in the adults of central China than in whites, blacks, and Hispanics [21]. The differentials may be attributed to geographic variation in sociodemographic characteristics, health literacy, and use of health care. The model indicated that sociodemographic characteristics, lifestyle, and obesity had significant direct relationships with CDH. Sociodemographic characteristics, lifestyle, and CDH were also indirectly related. The direct and indirect paths suggested that lifestyle was a dominant predictor of CDH.

The sociodemographic characteristics in the present study included three observed variables: age, educational level, and PCFMI. With regard to age, the paths indicated that older participants were prone to develop CDH probably because insulin resistance (IR) increased with age [45, 46]. IR is a common feature of prehypertension and prediabetes, which are early stages that can develop into HT and DM [47]. 70% of patients with type II diabetes have HT, which is primarily due to IR [48]. With regard to educational status, our results suggested that people who had higher education level had relatively higher risk of CDH. Similarly, previous studies have reported that educational status is a key variable that affects the prevalence of HT [49] and DM [50]. Education may play an important role in guarding against disease influenced by lifestyle. With regard to occupation, the results indicated that occupation could clearly predict CDH, and agricultural workers were more likely to obtain CDH probably because they were generally less educated than workers in other industries in China. This observation was consistent with previous findings. Their knowledge and understanding of HT and diabetes prevention were limited, which prevented them from adopting a healthy lifestyle. In addition, people with higher PCFMI were less likely to have CDH. People with low PCFMI likely focus on reducing expenses and increasing their income and pay less attention to their health compared with those with high PCFMI. Furthermore, they may engage in behaviour that can improve their income even if it is bad for their health.

In the present study, lifestyle included four observed variables: smoking, drinking, physical exercise, and psychosocial work intensity. The direct association between smoking and CDH is strong, and smokers likely develop CDH than non-smokers probably because smoking can increase IR [51, 52]. The direct association between drinking and CDH should be interpreted with caution. The relationship between alcohol consumption and increased risk of HT is clear in the latest study [15], whereas the relationship between alcohol consumption and DM is complex because the researchers must consider the volume and frequency of alcohol consumption. The amount of alcohol consumed may be more important than the frequency of alcohol consumption as a risk factor for DM [53]. However, our study simply divided the subjects into drinkers and non-drinkers. Previous epidemiological studies have shown that light-to-moderate alcohol consumption is associated with a lower risk for DM compared with abstention [54, 55]. The risk for heavy alcohol consumption was equal to or greater than that for abstainers [56, 57]. One review has suggested a U-shaped relationship between alcohol consumption and the risk of DM [58]. Our study suggests that physical exercise is negatively correlated with CDH, but their direct association is not particularly strong. Physical exercise has two main functions. On the one hand, moderate physical exercise can lead to the decrease of resistance around the blood circulation and decrease of cardiac output [59, 60]. On the other hand, moderate physical exercise can increase the sensitivity of endogenous insulin [61]. The association amongst DM, HT, and psychosocial work intensity has been highlighted in other studies. The hypothesis that psychosocial work intensity increases the risk of DM has not been confirmed in a meta-analysis [62]. However, Eriksson et al. have reported that work intensity might contribute to the development of DM [63]. Controversies about the relationship between psychological work intensity and HT are also found. For example, one study conducted in the Netherlands has suggested that psychological work intensity and HT have no significant relationship [64], whereas Liu et al. have found that psychological work intensity is associated with an increased risk of HT [65]. Determining the relationship between psychological work intensity and CDH is difficult. Our study found that the higher the work stress of adults, the higher the risk of CDH. However, in the future, accurately quantifying psychological work intensity and further verifying the relationship between psychological stress and CDH are necessary.

Our study indicated that salt intake had no significant relationship with CDH, which was not consistent with the results of previous study [66]. The reasons for this discrepancy in the result may be complex. There is no doubt that insulin resistance is closely related to hypertension and diabetes. Insulin resistance may lead to hypertension directly by affecting sodium retention, activating the sympathetic nervous system [67]. Insulin resistance leads to diabetes mainly because insulin resistance affects the distribution of glucose in muscle and fat, and weakens insulin to inhibit glucose output in the liver [68]. The key issue, however, is that the relationship between salt intake and insulin resistance is controversial [69, 70]. In addition, the inconsistent results among studies are also likely due to methodological differences, such as the use of various cut-off points for the amount of salt intake.

Our results also suggested that obesity was a significant predictor of CDH, which may be related to IR caused by obesity. IR is a common feature in patients with prehypertension and prediabetes [47]. Obesity is the leading cause of IR [71]. The mechanisms of obesity leading to IR are as follows: obesity is accompanied by the development of a chronic low-grade inflammation, which is promoted by expanding adipose tissue [72]. The expansion of fat mass characterised by adipocyte enlargement fuels the infiltration of macrophages into the adipose tissue [73]. The enhanced inflammatory trait of the adipose tissue instigates the production of cytokines, which contribute to the development of IR [74]. Anthropometry provides an alternative evaluation of obesity at lower cost. In our study, WC and WHR were found to be more strongly correlated with CDH than BMI. Several researchers have found limitations in the use of BMI and identified that WC and WHR were better predictors to show the correlations between obesity and IR than BMI [75, 76], which may explain our results.

In addition, the present study indicated that health knowledge had an indirect negative correlation with the prevalence of CDH, which was mediated by lifestyle or obesity. Therefore, access to health knowledge improves lifestyle changes and weight control, which can indirectly reduce the risk of CDH.

This study presents some limitations that should be considered. Firstly, although we used face-to-face interviews, all data were collected from a respondent-completed questionnaire; thus, responses may have a level of inherent inaccuracy or bias. Secondly, although we used a four-stage stratified sampling method, sampling errors are still inevitable. Finally, the present research may not have considered variables that could have possibly influenced the prevalence of CDH, such as family history of HT or DM.

## 5. Conclusions

At present, no literature has been found to study the relationship amongst sociodemographic characteristics, lifestyle, obesity, and CDH in adults by SEM. Our study shows that sociodemographic characteristics, lifestyle, and obesity are important influencing factors that have direct or indirect impact on CDH prevalence of adults. The relationship between salt intake and CDH needs further study in the future. Lifestyle variables (smoking, drinking, exercise, and work intensity) and obesity variables (BMI, WC, and WHtR) are relatively changeable. Therefore, in the future, these variables can be major targets for developing prevention strategies for CDH. In addition to strengthening the popularization of health knowledge, performing lifestyle and weight loss intervention in a differentiated way can be applied while developing health promotion program.

## Figures and Tables

**Table 1:** General characteristics of the survey participants and CDH.

Variable	*N* (%)	CDH *n* (%)	*χ* ^2^
Gender			18.48^*∗∗*^
Male	12214 (48.2)	400 (56.1)	
Female	13142 (51.8)	313 (43.9)	
Age (years)			386.08^*∗∗*^
18–39	10130 (40.0)	34 (4.8)	
40–59	8717 (34.4)	361 (50.6)	
≥60	6509 (25.6)	318 (44.6)	
Marital status			196.61^*∗∗*^
Unmarried	2012 (7.9)	77 (10.8)	
Married	21328 (84.1)	485 (68.0)	
Divorce/widowhood/separated	2016 (8.0)	151 (21.2)	
Education status			361.08^*∗∗*^
Illiterate	2807 (11.1)	232 (32.5)	
Primary school	7523 (29.7)	179 (25.1)	
Junior high school	8515 (33.6)	210 (29.5)	
High school	3368 (13.3)	45 (6.3)	
University or above	3143 (12.4)	47 (6.6)	
Occupation			29.60^*∗∗*^
Management	4943 (19.5)	194 (27.2)	
Professional	3923 (15.5)	109 (15.3)	
Business or services worker	8944 (35.3)	213 (29.9)	
Agricultural worker	7546 (29.7)	197 (27.6)	
PCFMI (RMB)			45.94^*∗∗*^
<1000	6074 (24.0)	208 (29.2)	
1000–1500	8004 (31.6)	274 (38.4)	
1500–2000	8639 (34.1)	165 (23.1)	
≥2000	2639 (10.4)	66 (9.3)	
Smoking			783.97^*∗∗*^
Yes	4384 (23.1)	402 (56.4)	
No	20972 (81.5)	311 (43.6)	
Drinking			131.80^*∗∗*^
Yes	6681 (26.3)	321 (45.0)	
No	18675 (73.7)	392 (55.0)	
Work intensity			3*e* + 03^*∗∗*^
High	1428 (5.6)	370 (51.9)	
Median	11429 (45.1)	277 (38.9)	
Low	12499 (49.3)	66 (9.3)	
Daily static behaviour time (h)			467.72^*∗∗*^
<4	16427 (64.8)	190 (26.7)	
≥4	8929 (35.2)	523 (73.3)	
Whether know salt consumption can affect health			144.55^*∗∗*^
Yes	15962 (63.0)	296 (41.5)	
No	9394 (37.0)	417 (58.5)	
Daily salt intake (g)			23.47^*∗∗*^
<6	1713 (6.8)	25 (3.5)	
6–12	2055 (8.1)	37 (5.2)	
12–18	8056 (31.8)	231 (32.4)	
>18	13532 (53.3)	420 (58.9)	
Physical exercise			19.71^*∗∗*^
Yes	4833 (19.1)	90 (12.6)	
No	20523 (80.9)	623 (87.4)	
Whether know the standard of daily salt intake			20.50^*∗∗*^
Yes	5277 (20.8)	100 (14.0)	
No	20079 (79.2)	613 (86.0)	
Whether know the standard of daily oil intake			5.37^*∗*^
Yes	4568 (18.0)	105 (14.7)	
No	20788 (82.0)	608 (85.3)	
WC			276.58^*∗∗*^
Normal	13528 (53.4)	162 (22.7)	
Abnormal	11828 (46.7)	551 (77.3)	
BMI (kg/m^2^)			682.87^*∗∗*^
<18.5	2304 (9.1)	29 (4.1)	
18.5∼23.9	15023 (59.3)	174 (24.4)	
24∼26.9	5816 (22.9)	290 (40.7)	
≥27	2213 (8.7)	220 (30.8)	
Whether know the risk standard of chronic diseases			33.43^*∗∗*^
Yes	7048 (27.8)	130 (18.2)	
No	18308 (72.2)	583 (81.8)	
WHtR			417.81^*∗∗*^
<*P*_25_	6421 (25.3)	58 (8.1)	
*P*_25_∼<*P*_50_	6307 (24.9)	72 (10.1)	
*P*_50_∼<*P*_75_	6553 (25.8)	202 (28.3)	
≥*P*_75_	6075 (24.0)	381 (53.5)	

^*∗*^ *P* < 0.05;^*∗∗*^*P* < 0.01.

**Table 2:** VIFs and correlation matrix for study variables (*N* = 25,356).

Variables	1	2	3	4	5	6	7	8	9	10	11	12	13	14	15	VIF
*Sociodemographic characteristics*																
(1) Age	1															1.33
(2) Education	−0.399^*∗*^	1														1.37
(3) Occupation	0.123^*∗*^	−0.173^*∗*^	1													1.07
(4) Income	−0.211^*∗*^	−0.075^*∗*^	−0.125^*∗*^	1												1.07

*Lifestyle*																
(5) Smoking	0.072^*∗*^	0.012	0.033^*∗*^	0.033^*∗*^	1											1.23
(6) Drinking	0	0.082^*∗*^	0.001	0.076^*∗*^	0.415^*∗*^	1										1.24
(7) Physical exercise	−0.114^*∗*^	0.173^*∗*^	−0.126^*∗*^	0.040^*∗*^	−0.044^*∗*^	0.007	1									1.08
(8) Work intensity	0.014^*∗*^	0.026^*∗*^	−0.085^*∗*^	−0.058^*∗*^	−0.112^*∗*^	−0.119^*∗*^	0.048^*∗*^	1								1.04
Health knowledge																
(9) Salt consumption can affect health	−0.164^*∗*^	0.210^*∗*^	−0.130^*∗*^	0.063^*∗*^	−0.015^*∗*^	0.039^*∗*^	0.185^*∗*^	0.026^*∗*^	1							1.23
(10) The standard of daily oil intake	−0.091^*∗*^	0.143^*∗*^	−0.100^*∗*^	0.007	−0.011	0.032^*∗*^	0.115^*∗*^	0.031^*∗*^	0.285^*∗*^	1						1.35
(11) The standard of daily salt intake	−0.085^*∗*^	0.231^*∗*^	−0.096^*∗*^	0.010	−0.032^*∗*^	−0.003	0.128^*∗*^	0.040^*∗*^	0.313^*∗*^	0.455^*∗*^	1					1.43
(12) The risk standard of chronic diseases	−0.107^*∗*^	0.180^*∗*^	−0.088^*∗*^	0.062^*∗*^	−0.015^*∗*^	0.025^*∗*^	0.171^*∗*^	0.037^*∗*^	0.271^*∗*^	0.292^*∗*^	0.274^*∗*^	1				1.19

*Obesity indicators*																
(13) BMI	0.044^*∗*^	−0.113^*∗*^	−0.122^*∗*^	0.018	0.053^*∗*^	0.054^*∗*^	−0.024^*∗*^	−0.053^*∗*^	−0.035^*∗*^	0.019	0.201^*∗*^	−0.027^*∗*^	1			1.22
(14) WC	0.099^*∗*^	0.120^*∗*^	−0.039^*∗*^	0.007	0.059^*∗*^	0.067^*∗*^	−0.033^*∗*^	−0.049^*∗*^	−0.030^*∗*^	−0.030^*∗*^	−0.125^*∗*^	−0.031^*∗*^	0.370^*∗*^	1		1.95
(15) WHtR	0.206^*∗*^	−0.186^*∗*^	−0.007	−0.028^*∗*^	0.014^*∗*^	0.011	−0.050^*∗*^	−0.025^*∗*^	−0.071^*∗*^	−0.053^*∗*^	−0.130^*∗*^	−0.057^*∗*^	0.356^*∗*^	0.804^*∗*^	1	3.04

^*∗*^ *P* < 0.05.

**Table 3:** Direct and indirect effects of obesity, lifestyle, health knowledge, and sociodemographic characteristics on CDH.

Variables	Path coefficient (95% CI)		
	Total	Direct	Indirect
Obesity	0.353^*∗∗*^ (0.321, 0.386)	0.353^*∗∗*^ (0.321, 0.386)	0
Health knowledge	−0.089^*∗∗*^ (−0.1032, −0.072)	0	−0.089^*∗∗*^ (−0.1032, −0.072)
Sociodemographic characteristics	0.315^*∗∗*^ (0.278, 0.352)	0.187^*∗∗*^ (0.157, 0.217)	0.128^*∗∗*^ (0.121, 0.135)
Lifestyle	0.784^*∗∗*^ (0.746, 0.824)	0.739^*∗∗*^ (0.711, 0.767)	0.045^*∗∗*^ (0.035, 0.057)

^*∗∗*^ *P* < 0.001 (two-tailed test).

## Data Availability

Materials included in the manuscript, excluding the relevant raw data, will be made freely available to any researchers who wish to use them for noncommercial purposes, while preserving any necessary confidentiality and anonymity.

## Supplementary Materials

Supplementary Materials Supplementary Table 1 presents the 15 observational variables in the measurement model.
