# Supplementary material for: Impact of Sociodemographic Characteristics, Lifestyle, and Obesity on Coexistence of Diabetes and Hypertension: A Structural Equation Model Analysis amongst Chinese Adults
Source: Int J Hypertens. 2021 Oct 25;2021:4514871. doi: 10.1155/2021/4514871 (PMC8560290; doi:10.1155/2021/4514871)
Supplement: Supplementary Materials — Supplementary Table 1 presents the 15 observational variables in the measurement model. [file 4514871.f1.docx]

**Supplementary Table 1 Assignment of study variables**

| **Variables** | **Value** |
| --- | --- |
| **Sociodemographic characteristics** |  |
| 1. Age (years) | 1 = 18-39, 2 = 40-59, 3 = ≥60. |
| 2. Educational status | 1 = Illiterate, 2 = Primary school, 3 = Junior high school,  4 = High school, 5 = University or above. |
| 3. Occupation | 1 = Management, 2 = Professional,  3 = Business or services worker, 4 = Agricultural worker. |
| 4.PCFMI (RMB) | 1 = < 1000, 2 = 1000-1500, 3 = 1500-2000, 4 = ≥ 2000. |
| **Lifestyle** |  |
| 1. Smoking | 1 = No, 2 = Yes. |
| 2. Drinking | 1 = No, 2 = Yes. |
| 3. Physical exercise | 1 = No, 2 = Yes. |
| 4. Work intensity | 1 = High, 2 = Median, 3 = Low. |
| **Health knowledge** |  |
| 1.Whether know salt consumption can affect health | 1 = No, 2 = Yes. |
| 2.Whether know the standard of daily salt intake | 1 = No, 2 = Yes. |
| 3.Whether know the standard of daily oil intake | 1 = No, 2 = Yes. |
| 4.Whether know the risk standard of chronic diseases | 1 = No, 2 = Yes. |
| **Obesity** |  |
| 1.BMI（kg/m^2^） | 1 = < 18.5, 2 = 18.5～23.9, 3 = 24～26.9, 4 = ≧ 27. |
| 2.WC | 1 = Normal, 2 = Abnormal. |
| 3.WHtR | 1 = < P_25_, 2 = P_25_～< P_50_,  3 = P_50_～< P_75_, 4 = ≧ P_75_. |

PCFMI: per capita family monthly income.
